# Clinical, Biological, and Treatment-Related Predictors of Central Nervous System Relapse in Diffuse Large B-Cell Lymphoma: A Retrospective Cohort Study

**DOI:** 10.3390/jcm15082866

**Published:** 2026-04-09

**Authors:** Cosmin-Daniel Minciuna, Dorina Minciuna, Angela-Smaranda Dascalescu, Amalia Titieanu, Vlad-Andrei Cianga, Ion Antohe, Ingrid-Andrada Vasilache, Catalin-Doru Danaila, Lucian Miron

**Affiliations:** 1 Grigore T. Popa University of Medicine and Pharmacy, 700115 Iasi, Romania; 2 Faculty of Medicine and Biological Sciences, ‘Ștefan cel Mare’ University, 720229 Suceava, Romania

**Keywords:** Diffuse Large B-Cell Lymphoma, overall survival, progression free survival, predictors, treatment response

## Abstract

**Background/Objectives**: Central Nervous System (CNS) relapse represents a severe and often fatal complication of Diffuse Large B-Cell Lymphoma (DLBCL). This study aimed to evaluate clinical, biological, and treatment-related factors associated with progression-free survival (PFS) until CNS relapse in patients with DLBCL. **Methods**: A retrospective cohort study was conducted using clinical data from adult DLBCL patients evaluated and treated at the Regional Institute of Oncology, Iași, Romania, between 2015 and 2023. Associations between clinical, biological, and treatment-related variables and CNS relapse were evaluated using univariate and multivariable Cox proportional hazards models, Fine–Gray competing-risk analyses, and propensity score-based methods to address confounding by indication for CNS prophylaxis. **Results**: Twenty-six CNS relapse events (6.3%) and 72 deaths without prior CNS relapse occurred over a median follow-up of 12 months. In the prespecified reduced multivariable Cox model, non-R-CHOP regimens (HR 4.57, 95% CI 1.67–12.52; *p* = 0.003) and high CNS-IPI scores (HR 4.70, 95% CI 1.14–19.46; *p* = 0.033) were independently associated with CNS relapse. The 20-month cumulative incidence of CNS relapse was 7.0% in the R-CHOP-like group versus 35.2% in the non-R-CHOP group (Gray’s test *p* < 0.001). Fine–Gray modeling confirmed the association for non-R-CHOP regimens (SHR 3.38, 95% CI 1.21–9.45; *p* = 0.02). Cell-of-origin subtype, double-expressor phenotype, and Ki-67 were not significantly associated with CNS relapse. **Conclusions**: High CNS-IPI and treatment with non-R-CHOP regimens independently predicted earlier CNS relapse. Future multicenter studies with molecular profiling are needed to refine CNS risk stratification.

## 1. Introduction

Diffuse Large B-cell Lymphoma (DLBCL) is the most common aggressive non-Hodgkin lymphoma in adults, and, despite substantial improvement in outcomes with rituximab-based immunochemotherapy, a subset of patients experience relapse or progression in the Central Nervous System (CNS). Population-based and clinical trial data consistently show that the cumulative incidence of CNS relapse in unselected DLBCL patients is 1.9% to 6.4% at 2–5 years after diagnosis and treatment, most commonly with rituximab-based immunochemotherapy [1,2,3,4,5]. The current literature cites the median time to CNS relapse as 7–15 months after initial diagnosis [1,2,6].

The risk is increased in patients with advanced disease stage, a high International Prognostic Index (IPI)/CNS-IPI score, multiple extranodal sites, and certain genetic mutations (e.g., *MYD88*, *CDKN2A*), and the CNS relapse rates can reach 8–12% at 2–5 years [1,4,5,7,8]. Furthermore, in elderly patients, the CNS relapse rates remain low (1.8–3%), but prognosis is especially poor [9].

CNS relapse is associated with severe neurological symptoms, rapid functional decline, and high rates of hospitalization and palliative care needs [10]. Intensive salvage therapies (e.g., high-dose methotrexate, stem cell transplantation) are often used, but only a minority of patients achieve durable remission [11,12]. In a large international cohort study conducted by El-Galaly et al. that included 291 patients with secondary CNS involvement treated with first-line immunochemotherapy, the authors reported a median post-CNS relapse overall survival (OS) of 3.9 months with a 2-year OS rate of 20% [11].

Tumor biology could capture intrinsic propensities for CNS tropism or treatment resistance. However, the relative and independent contributions of these features in real-world cohorts remain variably reported. For example, in the GOYA trial, the ABC subtype conferred a hazard ratio (HR) of 5.2 (95% CI: 2.1–12.9; *p* = 0.0004) for CNS relapse, independent of other risk factors [13]. When combined with a high CNS-IPI score, the 2-year CNS relapse rate reached 15.2%. Also, GCB patients had lower CNS relapse rates, and the cell-of-origin distinction improved risk stratification beyond clinical factors alone. Moreover, extranodal DLBCLs (testicular, breast, etc.) often have a non-GCB phenotype and *MYD88*/*CD79B* mutations, further increasing the CNS relapse risk [14,15].

Standard frontline therapy for most patients is R-CHOP or an R-CHOP-like regimen. For biologically aggressive disease, clinicians may escalate to intensive regimens (e.g., dose-adjusted R-DA-EPOCH or R-HyperCVAD). Whether such intensification modifies CNS relapse risk or improves post-CNS outcomes in routine practice is uncertain, mostly due to confounding elements such as patients’ baseline characteristics and disease phenotype. Literature data showed that R-CHOP and R-CHOP-like regimens were associated with low CNS relapse rates (1.9–6.4% at 2–5 years) [2,16,17,18], while intensive regimens did not reduce CNS relapses compared to R-CHOP in patients with synchronous CNS and systemic disease [19].

CNS prophylaxis remains another area of ongoing controversy. A small cohort study showed no statistically significant difference in CNS relapse rates between intrathecal (IT) chemotherapy and systemic high-dose methotrexate (HD-MTX) for prophylaxis in high-grade B-cell lymphoma, including DLBCL [20]. However, larger and meta-analytic studies suggest HD-MTX (with or without IT) is associated with a lower risk of CNS relapse than IT alone (Risk Ratio ~0.41; 95% CI: 0.24–0.68) [21,22].

The main purpose of this retrospective study was to determine the associations between key clinical, biological, and treatment-related predictors and both the risk of CNS relapse and progression-free survival until CNS relapse in a cohort of DLBCL patients from Romania.

## 2. Materials and Methods

### 2.1. Study Design and Setting

We performed a retrospective cohort study using the clinical database of adult patients diagnosed with DLBCL evaluated, treated, and followed at the Regional Institute of Oncology, Iași, Romania, between 2015 and 2023.

### 2.2. Eligibility Criteria

Inclusion criteria were represented by (i) patients with pathologically confirmed DLBCL; (ii) availability of routinely collected clinical, laboratory, and imaging data; and (iii) signed informed consent for medical data processing. We excluded patients with non-DLBCL histology, primary CNS lymphoma, incomplete medical records, or an undeterminable 26-month vital status assessment.

### 2.3. Variables and Data Collection

From electronic medical records, we extracted the following data for further analysis: demographics (age, sex), CNS-IPI, Eastern Cooperative Oncology Group—ECOG—performance status, Ki-67, and cell-of-origin subtype (ABC and GCB), and treatment/response variables (chemotherapy exposure, number of cycles, and treatment completion).

### 2.4. Treatment Exposures

Systemic therapy was classified a priori into two mutually exclusive categories:

− R-CHOP-like regimens: R-CHOP, R-CHOP-14, and R-mini-CHOP;
− Non-R-CHOP regimens: (dose-adjusted) R-EPOCH and R-HyperCVAD (intensive/salvage).

CNS prophylaxis was recorded in two ways: any CNS prophylaxis (intrathecal and/or systemic) and HD-MTX prophylaxis as a separate binary variable.

### 2.5. Outcomes and Time Scales

CNS relapse was defined by compatible neurologic symptoms and/or radiographic brain/leptomeningeal disease or positive cerebrospinal fluid (CSF) cytology/flow cytometry, adjudicated by treating clinicians.

Progression-free survival until CNS relapse (PFS until CNS) defined as time from index (start of frontline therapy) to CNS relapse/progression; patients without relapse were censored at last follow-up.

CSF sterilization denoted clearance of malignant cells on follow-up CSF sampling after therapy. For baseline descriptive comparisons, we analyzed the subset of patients who subsequently developed CNS relapse.

### 2.6. Statistical Analysis

Continuous variables were summarized as means (±standard deviation) and compared using *t*-tests or Wilcoxon tests as appropriate. Categorical variables were summarized as counts (percentages) and compared using Pearson’s χ^2^ or Fisher’s exact tests (two-sided α = 0.05). Baseline characteristics by chemotherapy group are displayed in Table 1.

Time-to-event outcomes were analyzed using Cox proportional hazards models. Univariate Cox regression was performed to evaluate the association between clinical and biological variables and the risk of CNS relapse.

A multivariable Cox model was constructed using a prespecified reduced set of clinically relevant variables (ECOG performance status, CNS-IPI, and chemotherapy regimen) to account for the limited number of events and optimize the events-per-variable (EPV) ratio.

An extended model including additional covariates was also explored and reported for completeness. Model assumptions were assessed using Schoenfeld residuals. Model fit was compared using Akaike Information Criterion (AIC).

Given the substantial number of deaths without prior CNS relapse, competing-risk analyses were performed using the Fine–Gray subdistribution hazard model. Death without CNS relapse was treated as a competing event, and subdistribution hazard ratios (SHRs) were estimated. Cumulative incidence functions were calculated for CNS relapse and competing mortality, and differences between groups were assessed using Gray’s test.

Propensity score (PS) methods were applied to address confounding by indication for CNS prophylaxis. A logistic regression model was used to estimate the probability of receiving prophylaxis based on baseline covariates.

Inverse probability of treatment weighting (IPTW) with stabilized and truncated weights was used to estimate adjusted hazard ratios. In addition, a sensitivity analysis using 1:1 nearest-neighbor propensity score matching (caliper 0.3) was performed.

Sensitivity analyses were conducted by sequentially excluding ECOG performance status and CNS-IPI from the multivariable model to assess robustness of effect estimates. An additional sensitivity analysis excluded patients with CNS involvement at diagnosis.

We evaluated multicollinearity between key predictors using generalized variance inflation factors (GVIFs) and correlation analyses. CNS-IPI was evaluated as both a categorical and continuous variable, and model performance was compared using AIC.

Cumulative incidence functions were estimated using competing-risk methods (Fine–Gray framework) and compared using Gray’s test. Kaplan–Meier curves were used for PFS, with group comparisons by log-rank test. A STROBE-compliant flow diagram was constructed to illustrate cohort selection.

All statistical analyses were performed in R version 4.5.3 (R Foundation for Statistical Computing, Vienna, Austria). Two-sided *p*-values < 0.05 were considered statistically significant.

## 3. Results

**Figure 1:**
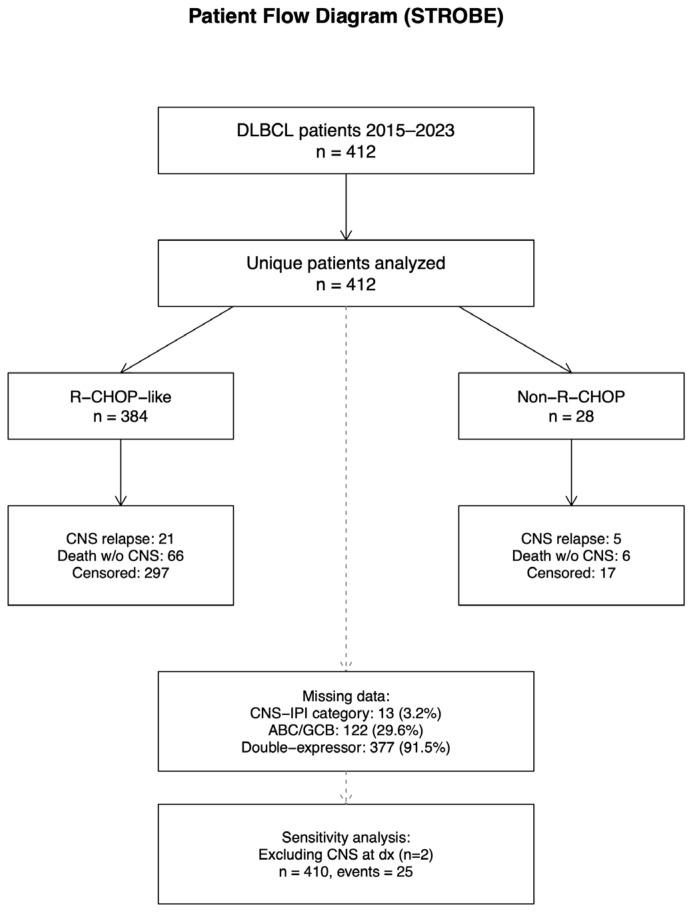
Patient flow diagram (STROBE).

A total of 412 unique patients were included in the study cohort, of whom 384 received R-CHOP-like regimens and 28 were treated with non-R-CHOP therapies. There were 26 CNS relapse events (6.31%) and 73 deaths overall, including 72 deaths occurring without prior CNS relapse (Figure 1).

Median follow-up was 12 months using the reverse Kaplan–Meier estimator, providing context for time-to-event interpretation in the presence of substantial early events. Two patients had CNS involvement at diagnosis (one in each chemotherapy group), and these patients were retained for the primary analysis but excluded in a prespecified sensitivity analysis to avoid the conflation of baseline CNS disease and secondary relapse.

Baseline characteristics of patients who developed CNS relapse, stratified by their chemotherapy regimen, are summarized in Table 1. Among these patients, 21 (80.8%) received an R-CHOP-like regimen (group 1), while five (19.2%) were treated with non-R-CHOP (intensive or salvage) chemotherapy (group 2).

The mean age at diagnosis was 58.1 ± 15.2 years in the R-CHOP-like group and 48.4 ± 18.6 years in the non-R-CHOP group, with no statistically significant difference (*p* = 0.229). The sex distribution was also comparable between groups, with males representing 42.9% and 60.0% of patients, respectively (*p* = 0.635).

Performance status did not differ significantly between treatment groups. However, patients treated with non-R-CHOP regimens more frequently presented with poor performance status (ECOG ≥ 3: 60.0% vs. 28.6%), whereas those receiving R-CHOP-like regimens more often had ECOG 0–1 (61.9% vs. 40.0%) (*p* = 0.564).

Similarly, CNS-IPI risk distribution showed a trend toward differences without reaching statistical significance (*p* = 0.055). All patients in the non-R-CHOP group were classified as intermediate risk (scores 3–4), whereas the R-CHOP-like group included patients across low (47.6%), intermediate (38.1%), and high (14.3%) risk categories.

With respect to biological characteristics, the ABC subtype was more frequent among patients treated with non-R-CHOP regimens (100% vs. 68.8%), while the GCB subtype was observed in the R-CHOP-like group only (31.2% vs. 0%); however, these differences were not statistically significant (*p* = 0.530 for both comparisons).

The frequency of the double-expressor phenotype was higher in the non-R-CHOP group (100% vs. 75.0%), although this difference did not reach statistical significance (*p* = 1.000). Similarly, the Ki-67 proliferation index did not differ significantly between groups (*p* = 0.433).

CNS involvement at diagnosis and CSF sterilization status were also comparable between groups, with no statistically significant differences observed (*p* = 0.192 and *p* = 1.000, respectively).

We performed a univariate Cox proportional hazards analysis that evaluated the association of clinical and biological predictors with the risk of CNS relapse, and the results are presented in Table 2.

Patients treated with non-R-CHOP regimens had a significantly higher risk of CNS relapse compared with those receiving R-CHOP-like therapy (HR 5.59, 95% CI 2.11–14.85, and *p* < 0.001). In contrast to the initial analysis, molecular subtypes were not significantly associated with CNS relapse risk. Neither the ABC subtype (HR 1.52, 95% CI 0.55–4.19, and *p* = 0.415) nor the GCB subtype (HR 0.66, 95% CI 0.24–1.81, and *p* = 0.415) showed a statistically significant effect. Similarly, the presence of the double-expressor phenotype was not associated with CNS relapse (HR 1.63, 95% CI 0.20–13.58, and *p* = 0.650).

Among clinical variables, poorer functional status remained an important predictor. Patients with an ECOG performance status ≥ 3 had a significantly increased risk of CNS relapse (HR 3.83, 95% CI 1.67–8.79, and *p* = 0.002), whereas the overall ECOG categorical variable was not significant (*p* = 0.223).

CNS-IPI was also associated with CNS relapse risk when analyzed as a continuous score, with each unit increase corresponding to a higher hazard (HR 1.51, 95% CI 1.11–2.06, and *p* = 0.010). Although the high-risk CNS-IPI category (5–6) showed a trend toward increased risk (HR 3.56, 95% CI 0.98–12.99, and *p* = 0.054), this did not reach statistical significance.

CNS prophylaxis was associated with a significantly increased risk of CNS relapse (HR 3.19, 95% CI 1.28–7.94, and *p* = 0.013). CNS involvement at diagnosis showed a non-significant trend toward higher risk (HR 5.73, 95% CI 0.78–42.30, and *p* = 0.087). Other variables, including age, sex, Ki-67 proliferation index, and molecular features, were not significantly associated with CNS relapse (all *p* > 0.05). Data on the cell-of-origin subtype was available in only 70.4% of patients, limiting its reliability for adjusted analyses. No significant association between molecular subtype and CNS relapse risk was observed in univariate analysis, and these variables were not retained in multivariable models due to missingness and limited statistical power.

ECOG performance status, CNS-IPI, and chemotherapy regimen remained key candidate predictors carried forward into multivariable modeling, consistent with the study’s central hypothesis and the original manuscript’s modeling approach.

### 3.1. Multivariable Cox Models for CNS Relapse

With 26 CNS relapse events, the full multivariable model (eight parameters) yielded an EPV of 3.2, suggestive of overfitting and unstable coefficient estimation. We therefore prespecified a reduced model including ECOG, CNS-IPI, and the chemotherapy group (five parameters; EPV 5.2) as the primary adjusted analysis, as shown in Table 3.

In the reduced model, high CNS-IPI (HR 4.70, 95% CI 1.14–19.46; *p* = 0.033) and non-R-CHOP regimens (HR 4.57, 95% CI 1.67–12.52; *p* = 0.003) were significantly associated with CNS relapse, with ECOG ≥3 showing a borderline trend (HR 2.43, 95% CI 0.94–6.26; *p* = 0.066).

The AIC of the full model (204.5) was lower than that of the reduced model (278.0), reflecting the different sample sizes used (complete cases with ABC subtype data vs. full cohort), and results should be interpreted with caution given the low EPV. The proportional hazards assumption was satisfied for the reduced model (Schoenfeld global *p* = 0.99).

### 3.2. Competing Risks

In competing-risk analyses treating death without prior CNS relapse as a competing event, event distribution was 314 censored, 26 CNS relapse, and 72 deaths without CNS relapse, demonstrating a meaningful competing event rate that could bias Kaplan–Meier-based cumulative incidence estimates if ignored.

**Figure 2:**
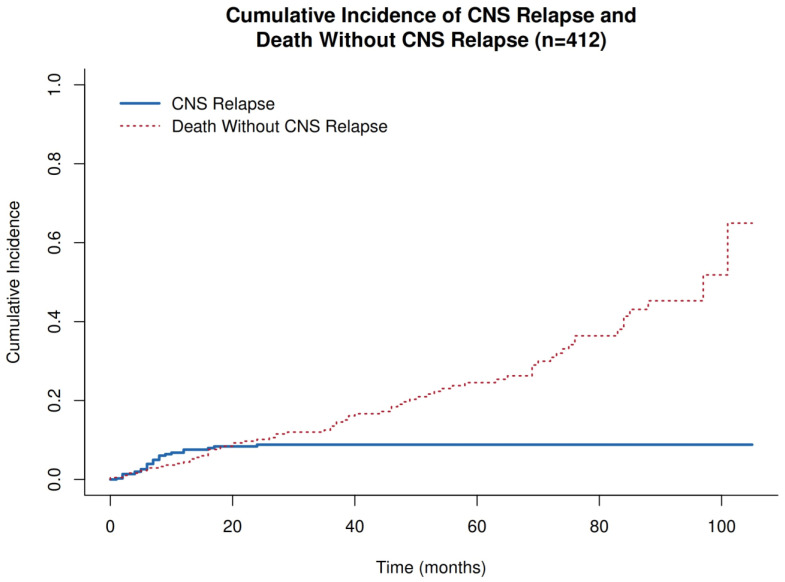
Cumulative incidence of CNS relapse and death without CNS relapse (overall cohort, n = 412).

The 20-month cumulative incidence of CNS relapse was 8.4% (95% CI 5.6–11.9%), while the 20-month cumulative incidence of death without CNS relapse was 9.3% (95% CI 6.2–13.1%), confirming that competing mortality was non-trivial in this real-world cohort (Figure 2).

**Figure 3:**
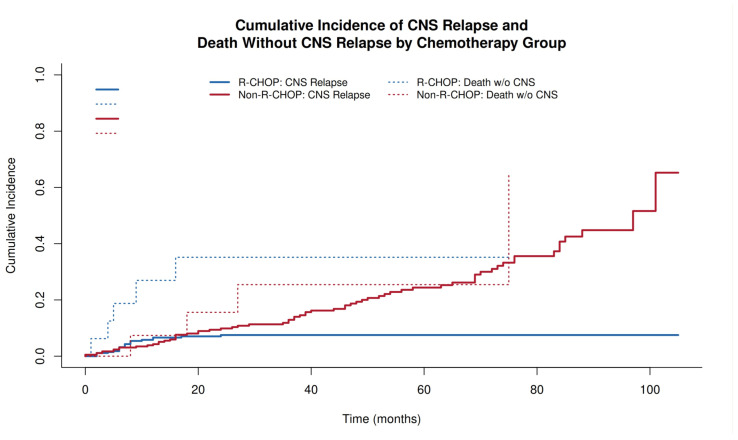
Cumulative incidence of CNS relapse and death without CNS relapse stratified by chemotherapy group (R-CHOP-like vs. non-R-CHOP).

By 20 months in the chemotherapy group, the cumulative incidence of CNS relapse was 7.0% in the R-CHOP-like group and 35.2% in the non-R-CHOP group, with Gray’s test indicating a significant difference for CNS relapse (*p* < 0.001), while the competing death incidence did not differ significantly (Gray’s test *p* = 0.31) (Figure 3).

In the Fine–Gray model fitted in complete cases (n = 283; 20 CNS events; and 54 competing deaths), non-R-CHOP remained independently associated with a higher CNS relapse risk (SHR 3.38, 95% CI 1.21–9.45; *p* = 0.02), as shown in Table 4. The SHRs for key covariates closely matched corresponding Cox HRs, supporting robustness of the main conclusions to competing-risk modeling (Table S1—comparison of standard Cox HR vs. Fine–Gray subdistribution hazard model).

### 3.3. Propensity Score Analyses for Prophylaxis

Because prophylaxis is preferentially administered to patients perceived as higher risk, we addressed confounding by indication using IPTW. The PS model identified age and the CNS-IPI score as strong predictors of prophylaxis receipt, as shown in Table 5.

Under IPTW with stabilized truncated weights (median 0.97; maximum 2.02), prophylaxis was not independently associated with CNS relapse (HR 1.75, 95% CI 0.63–4.86; *p* = 0.283), whereas non-R-CHOP (HR 3.96, 95% CI 1.33–11.83; *p* = 0.014) and ECOG ≥ 3 (HR 5.01, 95% CI 2.21–11.33; *p* < 0.0001) remained associated with increased CNS relapse risk, as shown in Table 6. These results indicate that the crude association between prophylaxis and CNS relapse observed in unadjusted models was driven by confounding by indication rather than a causal increase in relapse risk.

In PS matching (1:1 nearest neighbor, caliper 0.3), 28 matched pairs were obtained (four treated unmatched), with only five CNS relapse events in the matched cohort; prophylaxis remained non-significant (HR 3.28, 95% CI 0.40–26.98; *p* = 0.27), and the wide CI was interpreted as underpowered (Table 7). This direction is consistent with the IPTW results.

### 3.4. CNS-IPI Modeling and Multicollinearity Checks

Modeling CNS-IPI as a continuous score improved the model fit compared with categorical modeling (continuous: HR 1.64 per point, 95% CI 1.13–2.40; *p* = 0.010; AIC 275.0 vs. categorical AIC 278.0; and ΔAIC = 3.0 favoring continuous), as shown in Table 8.

Multicollinearity between ECOG and CNS-IPI was not prohibitive by GVIF (ECOG 1.48; CNS-IPI 1.48), although the correlation was moderate (Spearman ρ = 0.615), consistent with conceptual overlap but not indicating instability severe enough to preclude joint inclusion in reduced models, as shown in Table 9.

Sensitivity analyses were performed to assess the stability of the multivariable model by sequentially excluding ECOG performance status and CNS-IPI, as shown in Table 10.

In sensitivity models removing ECOG, high CNS-IPI retained significance (HR 4.25, 95% CI 1.15–15.74; *p* = 0.030). In sensitivity models removing CNS-IPI, ECOG ≥ 3 became strongly significant (HR 3.56, 95% CI 1.55–8.21; *p* = 0.003). Both predictors thus retain independent prognostic value when included together, supporting their joint inclusion in the primary reduced model.

### 3.5. Stratified and Adjusted Cox Models

A comparison between a stratified Cox model (with the chemotherapy group as a stratification variable) and an adjusted Cox model (with the chemotherapy group included as a covariate) is presented in Table 11. In the stratified model, ECOG and CNS-IPI estimates were similar to those from the adjusted model, with high CNS-IPI reaching significance (HR 4.81, 95% CI 1.16–19.87; *p* = 0.030). In the adjusted model (Table 11, non-R-CHOP was associated with a 4.6-fold higher CNS relapse hazard (HR 4.57, 95% CI 1.67–12.52; *p* = 0.003). Both approaches yielded consistent effect estimates for ECOG and CNS-IPI, supporting the robustness of these associations.

### 3.6. Sensitivity Excluding CNS Involvement at Diagnosis

After excluding the two patients with CNS involvement at diagnosis (n = 410; 25 CNS events), effect estimates were similar to the main analysis, with a non-R-CHOP HR of 3.45 (95% CI 0.84–14.17; *p* = 0.09), supporting the notion that baseline CNS involvement did not drive the principal treatment–regimen association, while acknowledging reduced precision due to fewer events, as shown in Table 12.

### 3.7. Progression-Free Survival Until CNS Relapse

**Figure 4:**
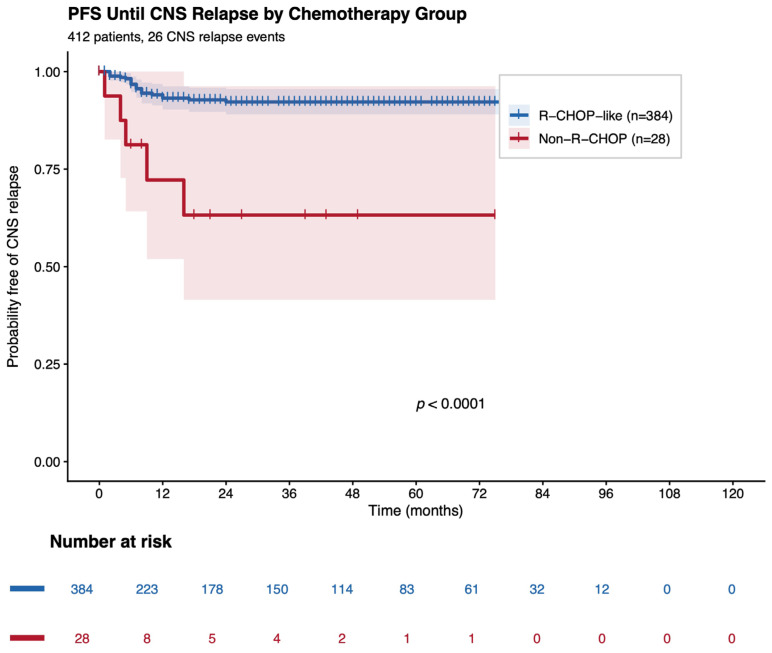
PFS until CNS relapse by chemotherapy group.

Progression-free survival until CNS relapse differed significantly by chemotherapy group (log-rank χ^2^ = 15.27, *p* < 0.001). In univariate Cox regression, non-R-CHOP regimens were associated with a substantially higher CNS relapse hazard. This association persisted in multivariable analyses, consistent with more aggressive disease biology and/or treatment selection in the non-R-CHOP subgroup, as shown in Figure 4.

## 4. Discussion

This retrospective cohort study evaluated clinical, biological, and treatment-related factors associated with CNS relapse and post-relapse outcomes in patients with DLBCL treated at a tertiary oncology center over an eight-year period.

Our findings indicate that patients treated with non-R-CHOP regimens had a substantially higher risk of CNS relapse compared with those receiving R-CHOP-like therapy. This association persisted even after adjustment. The non-R-CHOP group was characterized by a poorer performance status and intermediate CNS-IPI scores, suggesting that treatment selection reflected an already adverse baseline profile. Therefore, the higher relapse risk observed likely represents both disease-related aggressiveness and the limited ability of intensified therapy to overcome such unfavorable prognostic features.

The inferior outcomes observed in the non-R-CHOP group likely reflect an intrinsically aggressive tumor biology, potentially including subclinical CNS involvement at diagnosis. Intensified therapy may be insufficient to overcome these unfavorable features. Future NGS-based profiling may facilitate the early identification of patients at risk for CNS relapse, although the effectiveness of current therapeutic strategies in this setting remains uncertain

Previous studies have similarly shown that regimen escalation does not necessarily reduce CNS relapse incidence, particularly in patients with high-risk biological profiles or limited treatment tolerance. For example, in a phase II clinical trial conducted by Miyazaki et al., the authors evaluated the efficacy and safety of DA-EPOCH-R combined with high-dose methotrexate in a cohort of 47 patients diagnosed with CD5+ DLBCL [23]. The authors reported a 2-year PFS of 79%, a 2-year OS of 89%, as well as a CNS relapse rate of 9%. Moreover, the CNS relapse rate after DAR-EPOCH combined with HD-MTX is 7–9% [23,24,25,26]. Population-based data including R-HyperCVAD indicates CNS relapse rates of 2.3% at 3 years in high-risk DLBCL, lower than with R-CHOP plus intrathecal MTX (18.4%) or R-CHOP plus HD-MTX (6.9%) [21]. In the largest randomized trial, CNS relapse occurred in 4.0% of R-CHOP and 3.3% of DA-EPOCH-R patients, showing no significant difference [27]. Other studies confirm similar CNS relapse rates between these regimens [28,29]. Regarding the R-HyperCVAD regimen, data are limited, but available studies do not show a significant reduction in CNS relapse compared to R-CHOP or DA-EPOCH-R [30].

The incorporation of NGS-based profiling, including liquid biopsy approaches, may facilitate the early identification of high-risk mutations such as *MYD88*, enabling improved risk stratification and potentially guiding targeted therapeutic strategies. In this context, the addition of Bruton’s tyrosine kinase inhibitors, such as ibrutinib, to standard chemotherapy has been proposed in selected molecular subgroups, although this approach is not currently approved or reimbursed for this indication. Whether such targeted interventions can effectively reduce CNS relapse remains to be determined in prospective studies.

ECOG performance status emerged as an important clinical predictor of CNS relapse risk. Patients with ECOG ≥ 3 had a significantly increased risk in univariate analysis and showed a consistent trend toward higher risk in multivariable models, although this did not reach statistical significance after adjustment. This could point to the prognostic weight of functional impairment as a surrogate for both disease burden and systemic vulnerability.

The literature data showed that the baseline ECOG performance status (≥2) was consistently associated with significantly worse PFS and OS in DLBCL patients, independent of other risk factors. Multivariate analyses also showed that patients with a poor ECOG performance status have higher hazard ratios for both disease progression and death [31,32,33,34]. Moreover, combined risk models further improved risk stratification, identifying high-risk patients before first-line treatment who are more likely to experience refractory or relapsed disease [31]. The literature data confirmed that after CNS relapse or in secondary CNS lymphoma, a poor ECOG performance status at diagnosis was the strongest predictor of a poor outcome, often limiting the ability to receive CNS-directed therapy and correlating with inferior survival [35]. Also, in CAR-T therapy for relapsed or refractory DLBCL, the ECOG performance status remained an independent prognostic factor for both PFS and OS, even when accounting for comorbidities and other clinical variables [36,37].

High CNS-IPI scores were significantly associated with an increased risk of CNS relapse in multivariable analysis, confirming the index’s robustness in identifying patients at an elevated risk. In contrast, intermediate-risk categories did not retain statistical significance after adjustment, suggesting that prognostic discrimination is strongest at the extremes of the score distribution. This aligns with prior multicenter validation studies, which have reported variable predictive accuracy of CNS-IPI in intermediate-risk cohorts [5,38].

High CNS-IPI scores (4–6) were consistently linked to a significantly increased risk of CNS progression or relapse [5,39,40]. In large cohorts, 2-year CNS relapse rates were approximately 0.6–0.8% (low risk), 3.4–5.1% (intermediate risk), and 10.2–12.9% (high risk) [5,7,38,39]. High CNS-IPI scores also predicted a shorter time to CNS relapse and overall worse clinical outcomes [5,38,40]

Patients with high CNS-IPI scores had significantly shorter PFS compared to those with lower scores, reflecting both higher CNS and systemic relapse rates [5,38,40,41,42]. Also, high CNS-IPI was associated with reduced OS, as CNS relapse is a fatal complication with a poor prognosis [5,38,43].

In contrast to some prior reports, the cell-of-origin subtype (ABC vs. GCB) was not significantly associated with CNS relapse risk in our cohort. Moreover, due to missing data (available in only 70.4% of patients) and limited statistical power, these variables were not retained in multivariable models. Therefore, no definitive conclusions regarding the prognostic role of molecular subtype can be drawn from the present analysis. The literature data indicates that the ABC subtype had an HR of 5.2 for CNS relapse, and combining the ABC subtype with high CNS-IPI further increased the risk (2-year CNS relapse: 15.2% for high-risk group) [44,45]. While ABC DLBCL is linked to a higher CNS relapse risk, some authors suggest that, after CNS relapse, ABC patients may have a relatively improved overall survival compared to GCB, possibly due to differences in disease biology or response to salvage therapies [46].

CNS-directed prophylaxis appeared to be associated with a higher risk of CNS relapse in unadjusted analyses. However, this association was no longer significant after adjustment using propensity score-based methods (IPTW and matching), indicating that the initial signal was likely driven by confounding by indication. These findings suggest that prophylaxis was preferentially administered to higher-risk patients rather than exerting a causal detrimental effect, as demonstrated by some authors [47,48,49]. Several studies highlighted that HD-MTX prophylaxis significantly reduced the rate of isolated CNS relapse (3.1% vs. 14.6% at 3 years, *p* = 0.032) but did not reduce concomitant systemic CNS relapse and overall systemic relapse or improve PFS/OS in multivariable or propensity-matched analyses [48,50,51,52].

The Ki-67 proliferation index and double-expressor phenotype were not significantly associated with CNS relapse risk in either univariate or multivariable analyses. This may reflect limited statistical power and potential collinearity with established clinical risk factors such as CNS-IPI and the ECOG performance status. Nevertheless, these markers remain biologically relevant, as high proliferative activity and dual MYC/BCL2 expression are known to be predisposed to extranodal spread, including CNS involvement [44,53].

Our data collectively indicate that functional status and CNS-IPI are the most clinically relevant predictors of CNS progression and post-relapse survival in DLBCL. Patients exhibiting a high CNS-IPI or an ECOG score of ≥2 should be prioritized for CNS-directed prophylaxis, ideally with systemic high-dose methotrexate when practical, along with enhanced neurological surveillance. However, given the limitations of current clinical risk models, future efforts should focus on identifying novel predictive biomarkers, including molecular signatures derived from next-generation sequencing. Integrating genomic profiling into risk stratification may enable more precise identification of patients at the highest risk for CNS involvement and facilitate the development of biologically guided preventive strategies.

This study possesses multiple limitations intrinsic to its retrospective, single-center approach. The limited sample size constrains statistical power for identifying mild effects and prevents comprehensive subgroup analysis. Also, the small number of CNS relapse events (n = 26) resulted in wide confidence intervals, limiting statistical power and precision, and therefore, findings should be interpreted with caution. Key biological data were missing for a substantial proportion of patients, limiting the biological risk adjustment. Furthermore, despite the verification of proportional hazards assumptions, we cannot entirely dismiss time-dependent effects due to the restricted number of relapse episodes. The follow-up duration (median 12 months) may limit the capture of late CNS events, and further studies should focus on longer follow-up periods for these patients.

## 5. Conclusions

This study identifies non-R-CHOP chemotherapy regimens and high CNS-IPI scores emerging as the principal independent predictors of CNS relapse. Poor performance status (ECOG ≥ 3) showed a consistent association with increased risk, although it did not retain statistical significance after adjustment.

The apparent association between CNS-directed prophylaxis and higher relapse risk in unadjusted analyses was not confirmed after propensity score-based adjustment, supporting confounding by indication rather than a causal effect.

Future multicenter studies with larger cohorts and genetic profiling are necessary to corroborate these findings and enhance personalized strategies for CNS risk management in DLBCL patients.

Identification of targetable mutations, such as those involving the B-cell receptor pathway, may support the integration of targeted agents, including ibrutinib, into future preventive and therapeutic strategies.

## Figures and Tables

**Table 1:** Baseline clinical and biological characteristics according to chemotherapy regimen in DLBCL patients who developed CNS relapse.

Variable	Category	R-CHOP-Like Regimens (n = 21)	Non-R-CHOP Regimens (n = 5)	*p*-Value
Sex	Male	9 (42.9%)	3 (60.0%)	0.635
	Female	12 (57.1%)	2 (40.0%)	
Age (years)	Mean ± SD	58.1 ± 15.2	48.4 ± 18.6	0.229
ECOG performance status	0–1	13 (61.9%)	2 (40.0%)	0.564
	2	2 (9.5%)	0 (0%)	
	≥3	6 (28.6%)	3 (60.0%)	
CNS-IPI risk group	Low (1–2)	10 (47.6%)	0 (0%)	0.055
	Intermediate (3–4)	8 (38.1%)	5 (100%)	
	High (5–6)	3 (14.3%)	0 (0%)	
Cell of origin—ABC subtype	Positive (1)	11 (68.8%)	4 (100%)	0.530
	Negative (0)	5 (31.2%)	0 (0%)	
Cell of origin—GCB subtype	Positive (1)	5 (31.2%)	0 (0%)	0.530
	Negative (0)	11 (68.8%)	4 (100%)	
Double-expressor phenotype	Yes (1)	3 (75.0%)	3 (100%)	1.000
Ki-67 cut-off	≥75%	12 (57.1%)	2 (40.0%)	0.433
CNS involvement at diagnosis	Yes (1)	21 (100%)	4 (80.0%)	0.192
CSF sterilization	Yes (1)	4 (19.0%)	1 (20.0%)	1.000

Legend: R-CHOP-like regimens include R-CHOP, R-CHOP-14, R-CHOP +HD-MTX, and R-mini-CHOP; non-R-CHOP regimens include (DA)R-EPOCH and R-HyperCVAD. SD—standard deviation; ECOG—Eastern Cooperative Oncology Group; CNS-IPI—Central Nervous System–International Prognostic Index; ABC—activated B-cell-like subtype; and GCB—Germinal Center B-cell-like Subtype.

**Table 2:** Univariate Cox proportional hazards model for CNS relapse.

Variable	HR	95% CI	*p*-Value
Age	0.98	0.96–1.01	0.127
Sex (female)	0.95	0.44–2.05	0.894
Chemotherapy group (non-R-CHOP)	5.59	2.11–14.85	<0.001
ECOG (overall)	0.40	0.09–1.75	0.223
ECOG ≥ 3	3.83	1.67–8.79	0.002
CNS-IPI (categorical)	1.70	0.75–3.89	0.207
CNS-IPI high (5–6)	3.56	0.98–12.99	0.054
CNS-IPI (continuous score)	1.51	1.11–2.06	0.010
ABC subtype	1.52	0.55–4.19	0.415
GCB subtype	0.66	0.24–1.81	0.415
Double-expressor phenotype	1.63	0.20–13.58	0.650
Ki-67 ≥ 75%	1.35	0.31–5.93	0.694
CNS prophylaxis	3.19	1.28–7.94	0.013
CNS involvement at diagnosis	5.73	0.78–42.30	0.087

Legend: ECOG—Eastern Cooperative Oncology Group; CNS-IPI—Central Nervous System–International Prognostic Index; ABC—activated B-cell-like subtype; GCB—Germinal Center B-cell-like Subtype; HR—hazard ratio; CI—confidence interval.

**Table 3:** Full versus reduced multivariable Cox models for CNS relapse.

Covariate	Full Model HR (95% CI)	*p*-Value	Reduced Model HR (95% CI)	*p*-Value
ECOG 2 vs. 0–1	0.44 (0.09–2.22)	0.323	0.28 (0.06–1.33)	0.110
ECOG ≥ 3 vs. 0–1	3.02 (1.02–8.93)	0.046	2.43 (0.94–6.26)	0.066
CNS-IPI Intermediate vs. Low	2.86 (0.87–9.39)	0.082	1.59 (0.62–4.08)	0.331
CNS-IPI High vs. Low	4.52 (0.70–29.06)	0.112	4.70 (1.14–19.46)	0.033
Age (per year)	0.98 (0.94–1.01)	0.205	—	—
Prophylaxis	1.38 (0.38–5.04)	0.622	—	—
ABC Subtype	1.51 (0.52–4.35)	0.448	—	—
Non-R-CHOP	3.28 (0.99–10.91)	0.053	4.57 (1.67–12.52)	0.003

Legend: ECOG—Eastern Cooperative Oncology Group; CNS-IPI—Central Nervous System–International Prognostic Index; ABC—activated B-cell-like subtype; GCB—Germinal Center B-cell-like Subtype; HR—hazard ratio; and CI—confidence interval. Model characteristics—full model: 26 events/8 parameters; reduced model: 26 events/5 parameters.

**Table 4:** Fine–Gray subdistribution hazard model (n = 257, 20 CNS events, and 54 competing deaths).

Covariate	SHR	95% CI	*p*-Value
ECOG 2 vs. 0–1	0.42	0.07–2.40	0.33
ECOG ≥ 3 vs. 0–1	3.04	0.95–9.73	0.061
CNS-IPI Intermediate vs. Low	2.77	0.59–13.1	0.20
CNS-IPI High vs. Low	4.56	0.49–42.3	0.18
Age (per year)	0.98	0.93–1.02	0.33
Prophylaxis	1.40	0.26–7.51	0.69
ABC Subtype	1.53	0.57–4.12	0.40
Non-R-CHOP	3.37	1.21–9.44	0.021

Legend: SHR—subdistribution hazard ratio; CI—confidence interval; ECOG—Eastern Cooperative Oncology Group performance status; CNS-IPI—Central Nervous System–International Prognostic Index; and ABC—activated B-cell-like subtype.

**Table 5:** Propensity score model for receipt of CNS-directed prophylaxis.

Predictor	Coefficient	SE	z	*p*-Value
Intercept	−0.699	0.716	−0.98	0.329
Age (per year)	−0.058	0.013	−4.41	<0.001
ECOG 2 vs. 0–1	−0.969	0.558	−1.74	0.082
ECOG ≥ 3 vs. 0–1	−1.066	0.638	−1.67	0.094
CNS-IPI score (per point)	0.663	0.196	3.38	<0.001
Non-R-CHOP vs. R-CHOP-like	0.783	0.596	1.31	0.189

Legend: ECOG = Eastern Cooperative Oncology Group performance status; CNS-IPI = Central Nervous System–International Prognostic Index.

**Table 6:** IPTW-weighted Cox model for CNS prophylaxis (n = 412, 26 events).

Covariate	HR	95% CI	*p*-Value
Prophylaxis	1.75	0.63–4.86	0.283
Non-R-CHOP	3.96	1.33–11.83	0.014
ECOG 2	0.53	0.12–2.36	0.402
ECOG ≥ 3	5.01	2.21–11.33	<0.001

Legend: HR = hazard ratio; CI = confidence interval; and ECOG = Eastern Cooperative Oncology Group performance status.

**Table 7:** Propensity score-matched analysis for prophylaxis.

Parameter	Value
Matched pairs	28
Unmatched treated	4
Events in matched sample	5
Prophylaxis HR	3.28
95% CI	0.37–29.35
*p*-value	0.288

Legend: HR = hazard ratio; CI = confidence interval.

**Table 8:** Comparison of Cox models using CNS-IPI as continuous versus categorical predictor.

Covariate	Continuous CNS-IPI Model HR (95% CI)	*p*-Value	Categorical CNS-IPI Model HR (95% CI)	*p*-Value
CNS-IPI Score (per point)	1.64 (1.13–2.40)	0.010	—	—
CNS-IPI Intermediate vs. Low	—	—	1.59 (0.62–4.08)	0.331
CNS-IPI High vs. Low	—	—	4.70 (1.14–19.46)	0.033
ECOG 2 vs. 0–1	0.23 (0.05–1.09)	0.065	0.28 (0.06–1.33)	0.110
ECOG ≥ 3 vs. 0–1	1.93 (0.75–4.93)	0.171	2.43 (0.94–6.26)	0.066
Non-R-CHOP	4.77 (1.77–12.84)	0.002	4.57 (1.67–12.52)	0.003

Legend: CNS-IPI = Central Nervous System–International Prognostic Index; ECOG = Eastern Cooperative Oncology Group; HR = hazard ratio; and CI = confidence interval.

**Table 9:** Assessment of multicollinearity between ECOG, CNS-IPI, and chemotherapy group.

Variable	GVIF	Df	GVIF^(1/(2 × Df))
ECOG performance status	1.48	2	1.10
CNS-IPI category	1.48	2	1.10
Chemotherapy group	1.03	1	1.02

Legend: ECOG = Eastern Cooperative Oncology Group performance status; CNS-IPI = Central Nervous System–International Prognostic Index; GVIF = generalized variance inflation factor; and Df = degrees of freedom. Correlation analyses—Spearman correlation (CNS-IPI vs. ECOG): ρ = 0.615, *p* = 3.41 × 10^−44^; Chi-squared test of association: χ^2^ = 126.5, *p* = 2.16 × 10^−26^.

**Table 10:** Sensitivity analyses excluding ECOG or CNS-IPI from the Cox model.

Covariate	Model Without ECOG HR (95% CI)	*p*-Value	Model Without CNS-IPI HR (95% CI)	*p*-Value
CNS-IPI Intermediate vs. Low	1.64 (0.71–3.75)	0.244	—	—
CNS-IPI High vs. Low	4.25 (1.15–15.74)	0.030	—	—
ECOG 2 vs. 0–1	—	—	0.44 (0.10–1.93)	0.275
ECOG ≥ 3 vs. 0–1	—	—	3.56 (1.55–8.21)	0.003
Non-R-CHOP	5.79 (2.14–15.63)	<0.001	4.47 (1.67–12.00)	0.003

Legend: ECOG = Eastern Cooperative Oncology Group performance status; CNS-IPI = Central Nervous System–International Prognostic Index; ABC = activated B-cell-like subtype; HR = hazard ratio; CI = confidence interval.

**Table 11:** Comparison of stratified and adjusted Cox models for CNS relapse.

Covariate	Stratified Model HR (95% CI)	*p*-Value	Adjusted Model HR (95% CI)	*p*-Value
ECOG 2 vs. 0–1	0.28 (0.06–1.32)	0.107	0.28 (0.06–1.33)	0.110
ECOG ≥ 3 vs. 0–1	2.39 (0.93–6.17)	0.071	2.43 (0.94–6.26)	0.066
CNS-IPI Intermediate vs. Low	1.57 (0.61–4.05)	0.348	1.59 (0.62–4.08)	0.331
CNS-IPI High vs. Low	4.81 (1.16–19.87)	0.030	4.70 (1.14–19.46)	0.033
Non-R-CHOP	—	—	4.57 (1.67–12.52)	0.003

Legend: ECOG = Eastern Cooperative Oncology Group performance status; CNS-IPI = Central Nervous System–International Prognostic Index; HR = hazard ratio; and CI = confidence interval.

**Table 12:** Sensitivity analysis excluding CNS involvement at diagnosis (n = 397, 25 events).

Covariate	HR	95% CI	*p*-Value
ECOG 2 vs. 0–1	0.28	0.06–1.32	0.107
ECOG ≥ 3 vs. 0–1	2.39	0.90–6.31	0.079
CNS-IPI Intermediate vs. Low	1.57	0.61–4.03	0.347
CNS-IPI High vs. Low	4.65	1.12–19.27	0.034
Non-R-CHOP	4.46	1.50–13.31	0.007

## Data Availability

The datasets are available from the correspondent authors upon reasonable request due to local policies.

## Supplementary Materials

The following supporting information can be downloaded at: https://www.mdpi.com/article/10.3390/jcm15082866/s1, Table S1. Comparison of Standard Cox HR vs. Fine–Gray Subdistribution Hazard Model.

## Abbreviations

The following abbreviations are used in this manuscript:

ABC	Activated B-cell–like
CAR-T	Chimeric Antigen Receptor T-cell
CI	Confidence Interval
CNS	Central Nervous System
CNS-IPI	Central Nervous System–International Prognostic Index
CSF	Cerebrospinal Fluid
CT	Computed Tomography
DA	Dose-Adjusted
DA-R-EPOCH	Dose-Adjusted Rituximab, Etoposide, Prednisone, Oncovin (vincristine), Cyclophosphamide, Hydroxydaunorubicin (doxorubicin)
DLBCL	Diffuse Large B-Cell Lymphoma
ECOG	Eastern Cooperative Oncology Group
GCB	Germinal Center B-cell–like
HD-MTX	High-Dose Methotrexate
HIV	Human Immunodeficiency Virus
HR	Hazard Ratio
IPI	International Prognostic Index
IT	Intrathecal
LDH	Lactate Dehydrogenase
MTX	Methotrexate
OS	Overall Survival
PET	Positron Emission Tomography
PET-CT	Positron Emission Tomography–Computed Tomography
PFS	Progression-Free Survival
R-CHOP	Rituximab, Cyclophosphamide, Hydroxydaunorubicin (doxorubicin), Oncovin (vincristine), Prednisone
R-CHOP-14	R-CHOP Given Every 14 Days (dose-dense)
R-mini-CHOP	Reduced-dose R-CHOP
R-EPOCH	Rituximab + Etoposide, Prednisone, Oncovin (vincristine), Cyclophosphamide, Hydroxydaunorubicin (doxorubicin)
R-HyperCVAD	Rituximab + Hyper-CVAD (hyperfractionated Cyclophosphamide, Vincristine, Adriamycin/doxorubicin, Dexamethasone; alternating with high-dose MTX/cytarabine)
SD	Standard Deviation
