# Supplementary material for: Clinical, Biological, and Treatment-Related Predictors of Central Nervous System Relapse in Diffuse Large B-Cell Lymphoma: A Retrospective Cohort Study
Source: J Clin Med. 2026 Apr 9;15(8):2866. doi: 10.3390/jcm15082866 (PMC13116589; doi:10.3390/jcm15082866)
Supplement: Supplementary file 1 [file jcm-15-02866-s001.zip › jcm-4187929-supplementary.pdf]

Table S1. Comparison of Standard Cox HR vs Fine–Gray Subdistribution Hazard Model

Covariate	Cox HR	Fine–Gray SHR	Difference
ECOG 2	0.44	0.42	0.02
ECOG ≥3	3.02	3.04	0.02
CNS-IPI Intermediate	2.86	2.77	0.09
CNS-IPI High	4.52	4.56	0.04
Age	0.98	0.98	0.00
Prophylaxis	1.38	1.40	0.02
ABC	1.51	1.53	0.02
Non-R-CHOP	3.28	3.37	0.09
